# Role of breast magnetic resonance imaging in predicting malignant invasion of the nipple-areolar complex: Potential predictors and reliability between inter-observers

**DOI:** 10.1097/MD.0000000000007170

**Published:** 2017-07-14

**Authors:** Chun-Ying Liao, Yu-Ting Wu, Wen-Pei Wu, Chih-Jung Chen, Hwa-Koon Wu, Ying-Jen Lin, Shou-Tung Chen, Dar-Ren Chen, Chi-Wei Lee, Shu-Ling Chen, Shou-Jen Kuo, Hung-Wen Lai

**Affiliations:** a Departemnt of Radiology, Changhua Christian Hospital, Changhua; b Department of Surgery, Ministry of Healthy and Welfare Sinying Hospital, Tainan; c Comprehensive Breast Cancer Center, Changhua Christian Hospital, Changhua; d School of Medicine, National Yang Ming University, Taipei; e Tumor Center; f Department of Surgical Pathology, Changhua Christian Hospital, Changhua; g School of Medicine, Chung Shan Medical University, Taichung; h Department of Medical Technology, Jen-Teh Junior College of Medicine, Nursing and Management, Miaoli, Taiwan.

**Keywords:** breast cancer, diagnostic accuracy, evaluation, magnetic resonance imaging, nipple-areolar complex

## Abstract

In this study, we assessed the diagnostic accuracy of breast magnetic resonance imaging (MRI) for evaluation of malignant invasion of the nipple-areolar complex (NAC).

Patients with primary operable breast cancer who underwent preoperative breast MRI and received surgery during January 2011 to December 2013 were collected. The accuracy and potential factors of MRI in predicting nipple invasion were evaluated by comparing preoperative MRI with postoperative histopathologic findings. The consistency of interobservers’ variances across different radiologists was also compared.

Totally, 704 patients were enrolled in this study, and 56 (8%) patients have pathologic NAC invasion. Several MRI factors were potential predictors of nipple invasion. Only unilateral nipple enhancement on MRI was the most significant independent predictor of NAC involvement in multivariate analysis. The statistical measures, such as sensitivity, specificity, positive predictive value (PPV), negative predictive value (NPV), and the accuracy of breast MRI were 71.4%, 81.6%, 25.2%, 97.1%, and 80.8%, respectively, in one investigator and 78.6%, 88.1%, 36.4%, 97.9%, and 87.4%, respectively, in the other investigator.

MR images showed acceptable accuracy and impressive NPV, but low PPV in evaluation of malignant NAC invasion preoperatively. MRI finding of unilateral nipple enhancement was the most significant predictor of NAC involvement.

## 1 Introduction

Preoperative prediction of nipple-areolar complex (NAC) invasion is necessary for adequate surgical planning so that the risk for occult nipple invasion or breast cancer recurrence can be minimized. In patients when mastectomy was indicated and preoperative evaluation showed no sign of NAC invasion, the “nipple-sparing mastectomy (NSM)” could be adopted instead of conventional mastectomy.^[1,2]^ Clinical or pathologic factors, such as tumor size, proximity to the NAC, multifocality and centrality of the tumor have been shown to be associated with occult invasion of nipple.^[3–5]^ However, these clinicopathologic factors may not be correctly predicted pre-operatively or only available after surgery.

Conventional diagnostic imaging modalities such as mammography and sonography have been shown to have value in predict NAC involvement.^[6]^ However, mammography is less accurate at detecting breast cancer in women with high breast density, and also often fails to reveal the retroareolar mass because it is difficult to differentiate it from the normal nipple structures or from normal retroareolar glandular density. Sonographic images usually depict nipples with posterior acoustic shadowing. Even when a malignant mass is found, it is difficult to evaluate the malignant invasion of the NAC. New image with better diagnostic value to predict NAC invasion is needed.

Contrast-enhanced dynamic magnetic resonance imaging (MRI) has been shown to be a useful imaging modality for the diagnosis of breast cancer. MRI, owing to the high morphological resolution and different enhancement patterns, makes it able to differentiate between normal or benign mammary tissue to malignant breast cancer.^[5,7]^ MRI of breast has been reported to have value in estimating tumor size,^[8,9]^ and been reported to have high sensitivity in detecting occult breast lesions. Because of these characteristics, breast MRI might be a powerful tool for assessing occult nipple invasion.

Some studies had focused on using MRI in prediction of NAC invasion^[10–12]^; however, the results were quite varied. The sensitivity was reported ranged from 28% to 100%, and specificity ranged from 22% to 100% according to different study groups.^[4,11–18]^ This wide variation of accuracy of breast MRI in prediction of NAC invasion might be because of the lack of objective criteria for diagnosing NAC invasion, and inconsistency of subjective qualitative opinion between radiologists. The reliability of interobservers between different radiologists in interpretation of NAC invasion in MR images was rarely discussed or tested. Owing to these reasons, the role of breast MRI in estimate NAC invasion before surgery remained controversial and related study was needed. We hypothesized that the lack of objective criteria for diagnosing NAC invasion, and inconsistency of subjective qualitative opinion between radiologists were the main factors affecting the diagnostic accuracy of breast MRI.

The aim of this study was to investigate the clinicopathologic factors and diagnostic accuracy of breast MRI for the assessment of malignant invasion of the NAC by comparing preoperative MR images with postoperative histopathologic findings. Some potential MR image factors were tested for the diagnosis of NAC invasion. The diagnostic accuracy of breast MRI and the consistency of interobservers’ variances across different radiologists would also be compared, and tested.

## 2 Materials and methods

### 2.1 Patients

**Figure 1:**
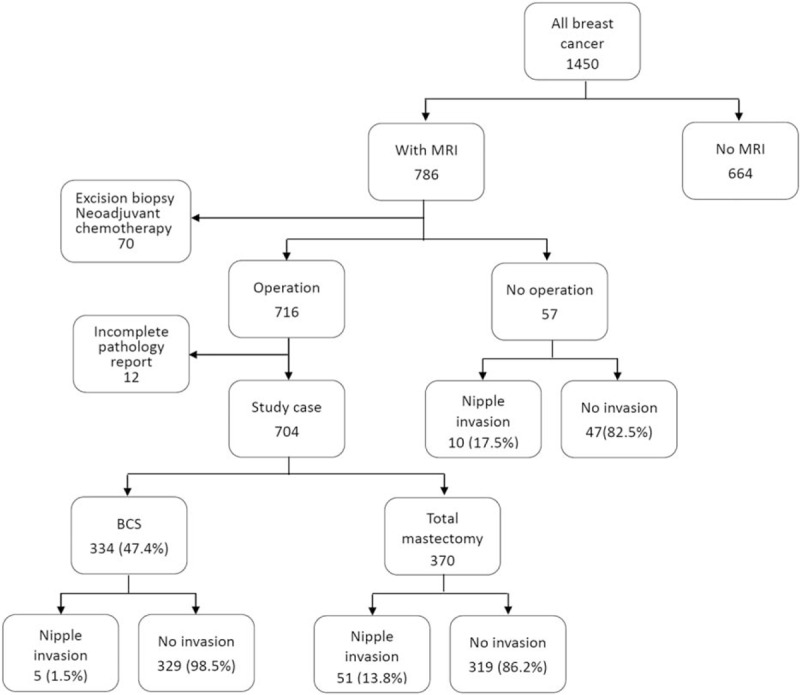
Flow chart of patients’ management in present study.

To evaluate the diagnostic accuracy of breast MRI in the prediction of NAC invasion of breast cancer, a retrospective study was conducted. Patients with primary operable breast cancer who underwent preoperative breast MRI and received surgery at the Changhua Christian Hospital (CCH), a tertiary medical center at central Taiwan, during the period of January 2011 to December 2013 were selected from the hospital's surgical database. The exclusion criteria included patients received excisional biopsy surgery with primary tumor removed before definite cancer operation, those who had received neoadjuvant chemotherapy, and patients whose detailed data were not available (Fig. 1). Preoperative MR images were evaluated and reported by the principal radiologist (HKW). The accuracy of MRI in predicting nipple invasion was evaluated by comparing preoperative MR images with postoperative histopathologic findings.

Data collection of this study was performed by a specially trained nurse (SLC) and the correctness of the data was checked by the principal investigator (HWL). This study was approved by the institutional review board (IRB) of the Changhua Christian Hospital (IRB No. 140404). Owing to the retrospective and chart review nature of this study, the ethics committees (IRB) in our hospital decided no written or verbal informed consent was needed by the participants. Patient records/information was anonymized and de-identified before analysis.

### 2.2 Clinicopahtologic and radiologic factors

The following clinicopathologic factors were retrospectively collected from medical records: age, tumor size, nipple invasion, lymph node (LN) metastasis, multifocality, margin status, grade, histologic types, stage, hormonal status, and operative method. Breast MRI examinations were retrospectively reviewed and recorded for tumor size, tumor-nipple distance, LN metastasis, nipple invasion or retraction, periareolar skin thickening, NAC enhancement, relationship to the subareolar mass, malignant mass pattern, thickness of NAC enhancement, and multifocal/multicentric lesion. In present study, subareolar (retroareolar) area is defined as that within 1 cm of the NAC.

### 2.3 Interobserver variance

To evaluate the interobserver variance between radiologists, another experienced breast radiologist (CYL), who was blinded to the final histopathologic diagnosis, was asked to report the related MRI findings and predict of NAC invasion. Concordance between the preoperative prediction of NAC invasion by breast MRI and the final pathology result was compared between different radiologists for evaluation of the diagnostic accuracy between radiologists. Statistical analysis for interobservers reliability (e.g., kappa statistics) was performed in this study. MR images analyzed for potential predictors as mentioned earlier were also compared between 2 observers for evaluation of the discrepancy between different radiologists, and positive predictive valued of each potential predictor.

### 2.4 Breast MRI protocol and prediction of NAC invasion

The MRI protocol is described in our previous study,^[8]^ and the data reported in the current analysis also include the patient data reported in the earlier publication. Briefly, a Siemens (Verio) 3.0 Tesla magnet MR imaging was used. All patients were imaged with both breasts placed into a dedicated 16-channel breast coil in the prone position. Both breasts were examined with a 60-second interval between each dynamic phase image in the transverse plane. A commercially available MRI computer aid diagnosis (CAD) system (DynaCAD Version 2.1 for Breast MRI [Invivo, Gainesville, FL]) was used to help analyzing MR images. The whole breast MR images were interpreted by experienced, board-certified radiologists specializing in breast imaging (HKW and CYL).

### 2.5 Definition of potential predictors of NAC invasion on MRI

Radiological factors analyzed in this study included 8 potential predictors (nipple inversion or retraction, periareolar skin thickening, NAC enhancement, relationship to the subareolar mass, malignant mass pattern, thickness of NAC enhancement, tumor-nipple distance, and tumor size), and the definition of these MR image factors were summarized briefly according to previous studies.^[8,11]^ Nipple inversion or retraction was evaluated using axial T1-weighted and fat-suppressed T2-weighted images. A complete loss of the normal nipple tip was considered as nipple inversion. A partial loss of the normal nipple tip was considered as nipple retraction. Periareolar skin thickening in the NAC was evaluated by comparing the contralateral NAC using the fat-suppressed T2-weighted images and contrast-enhanced T1-weighted images. Contrast-enhanced MRI was used for the evaluation of NAC enhancement and was measured for thickness of NAC enhancement.^[11]^ The enhancement characteristics of the nipple were evaluated on axial and sagittal reconstruction images. The relationship between enhancement of the NAC and the mass was evaluated and classified as a continuous or discontinuous pattern. If the tubular enhancing lesion between the mass and the NAC was depicted, it was considered as a continuous pattern. Thickness of the NAC enhancement was measured from the tip of the nipple to the enhancing lesion, and thickness of the NAC enhancement >3 mm was considered as positive enhancement. Using contrast-enhanced subtraction MRI, the tumor to nipple distance was measured from the mass closest to the base of the nipple. The enhancement pattern of the malignant mass was classified as mass or non-mass-like enhancement. The maximum diameter of the malignant mass was measured as tumor size,^[8]^ and, if there were multiple masses, the diameter of the largest mass was measured. The interpretation principle for measurement of tumor size by MRI was based on using a commercially available MRI CAD system with computer-based tumor segmentation in volume rendering data set by DynaCAD Version 2.1 for Breast MRI (Invivo, Gainesville, FL). For avoiding under estimate the tumor volume due to blooming effect and early peri-ductal enhancement, the result was manipulated by experienced radiologist after computer-based tumor segmentation.

### 2.6 Definition of sensitivity, specificity, and accuracy of breast MRI

The preoperative image findings were compared with the histopathologic findings to assess the sensitivity, specificity, positive predictive value (PPV), negative predictive value (NPV), and accuracy of MRI in detecting malignant invasion of the NAC. A true-positive (TP) diagnosis was defined in patients with preoperative MRI findings and histopathologic findings indicative of nipple invasion. A true-negative (TN) diagnosis was defined in patients with preoperative MRI findings showing no nipple invasion and histopathologic findings positive for a benign lesion. A false-negative (FN) was defined in patients with preoperative MRI findings showing no nipple invasion and histopathologic findings positive for a malignant lesion. A false-positive (FP) was defined in patients with preoperative MRI findings showing nipple invasion and histopathologic evidence of a benign lesion.

Sensitivity was calculated by dividing the number of true positives by the sum of total true-positives and false-negatives, that is, sensitivity = TP/(TP + FN). Specificity was calculated by dividing the number of true negatives by the sum of true negatives and false-positives, that is, specificity = TN/(TN + FP). PPV was calculated by dividing the total number of true positives by the sum of true-positives and false-positives, that is, PPV = TP/(TP + FP). NPV was calculated by dividing the total number of true negatives by the sum of true-negatives and false-negatives, that is, NPV = TN/(TN + FN). Accuracy was calculated by dividing the total number of all true-positives and true-negatives by the sum of all indicators, that is, accuracy = TP + TN/(TP + FN + TN + FP).

**Figure 2:**
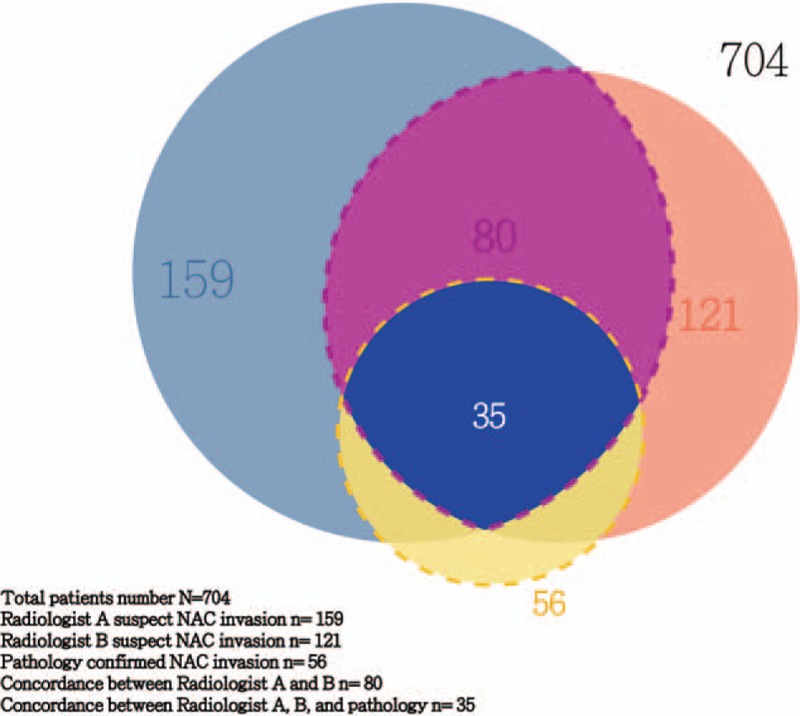
Union of positive findings in radiologist A, radiologist B, and pathologist.

The PPV _C(+)P(+)_ of each potential factor in each radiologist was calculated by dividing the total number of true-positives MR images findings in each radiologist (C(+)P(+)) by the sum of union of suspect NAC invasion in MRI in the 2 radiologists and positive results in pathologist (Fig. 2), that is, PPV _C(+)P(+)_ = C(+) P(+)/union of C_A_(+), C_B_(+), and (P)(+) (C: clinical, P: pathological).

### 2.7 Statistical analysis

Differences in means of continuous variables were tested by the Student *t* test and are reported as means ± standard deviation (SD). The *χ*^2^ test was used to assess the associations between nipple involvement and patients’ clinicopathologic factors or MRI findings. Significant variables in the univariate analyses were then included in a multivariate regression model to identify the most important factors. Cox proportional-hazards analysis was used to determine the relative contribution of tumor characteristics, patient characteristics, and MRI findings to the prediction of NAC invasion. Receiver-operating characteristic (ROC) curve tests were used in present study focusing on the diagnostic performance of MRI findings in predicting NAC invasion. Statistical analysis with kappa statistics was performed for test of interobservers’ reliability by package “irr” of R 3.2.2. A *P* value of <.05 was considered to indicate statistical significance. All statistical analyses were performed with the statistical package Statistical Product and Service Solutions (SPSS) for Windows (Version 19.0, SPSS Inc, Chicago, IL).

## 3 Results

**Figure 3:**
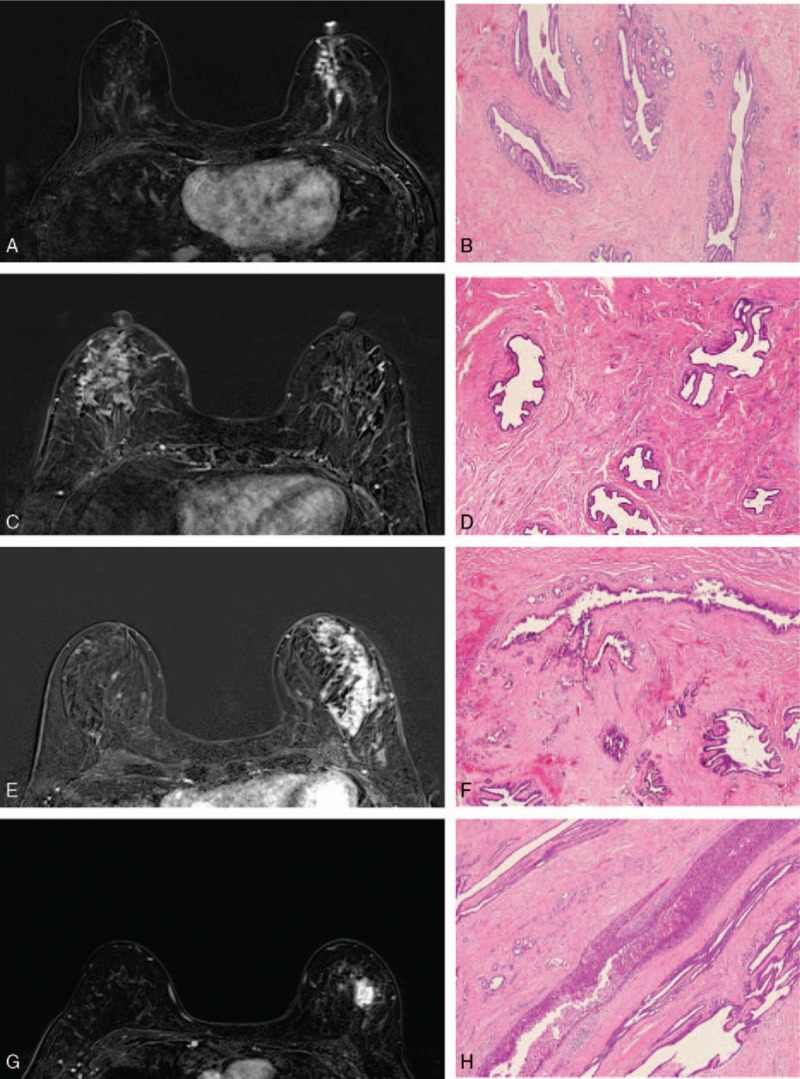
(A) A 53-year-old female diagnosed breast cancer, subtraction images of post gadolinium T1-weighted magnetic resonance imaging, axial view, showed non-mass like enhancement lesion in lower inner quadrant of left breast, in the central location, and size >2 cm, associated with unilateral nipple areolar complex (NAC) enhancement, continuous relationship between enhancement of the NAC and the lesion, periareolar skin thickening, and thickness of NAC enhancement >3 mm. Nipple areolar complex invasion was impressed from the magnetic resonance imaging findings. (B) Histopathologic samples stained with hematoxylin and eosin (40×) showed Paget cells in epidermis of nipple and ductal carcinoma in situ in lactiferous duct, confirmed the diagnosis of NAC invasion. (C) A 57-year-old female diagnosed breast cancer, subtraction images of post-gadolinium T1-weighted magnetic resonance imaging, axial view, showed non-mass-like enhancement lesion in the lower outer quadrant of right breast with the same finding as 1A, and NAC invasion was impressed from the magnetic resonance imaging findings. (D) Histopathologic samples stained with hematoxylin and eosin (40×) showed no tumor involvement of nipple and lactiferous duct, a false-positive MRI diagnosis for NAC invasion. (E) Another 57-year-old female diagnosed breast cancer, subtraction images of post gadolinium T1-weighted magnetic resonance imaging, axial view, showed non-mass-like enhancement lesion in upper outer quadrant of left breast, >2 cm in size, in the peripheral location, 3.1 cm distance to nipple (not shown in this figure), associated with discontinuous relationship between NAC and the lesion, no abnormal NAC enhancement, no periareolar skin thickening, and thickness of NAC enhancement <3 mm. Negative NAC invasion was impressed from the magnetic resonance imaging findings. (F) istopathologic samples stained with hematoxylin and eosin (40×) showed ductal carcinoma in situ in the subareolar tissue and the lactiferous duct, a false-negative MRI diagnosis for NAC invasion. (G) A 60-year-old female diagnosed breast cancer, subtraction images of post-gadolinium T1-weighted magnetic resonance imaging, axial view, showed a mass lesion in upper outer quadrant of left breast, >2 cm in size, in the peripheral location, 3.3 cm distance to nipple (not shown in this figure), with discontinuous relationship between NAC and the lesion, no abnormal NAC enhancement, no periareolar skin thickening, and thickness of NAC enhancement <3 mm. Negative NAC invasion was impressed from the magnetic resonance imaging findings. (H) Histopathologic samples stained with hematoxylin and eosin (40×) showed no tumor involvement of nipple and lactiferous duct, confirmed the diagnosis of negative NAC invasion.

A total of 704 patients with primary operable breast cancer fulfilled the inclusion and exclusion criteria. Of them, 334 (47.4%) received partial mastectomy and 370 (52.6%) received total mastectomy. Of the 370 patients who received total mastectomy, 154 (41.6%) underwent NSM. Among the 334 (47.4%) of patients who received partial mastectomy, 5 (1.5%) of them were found to have NAC invasion and received excision of the NAC. In total, 56 (8.0%) patients had histopathologic evidence of breast cancer with nipple invasion (Fig. 3). The demographic and image-related factors are summarized in Table 1 .

**Table 1:**
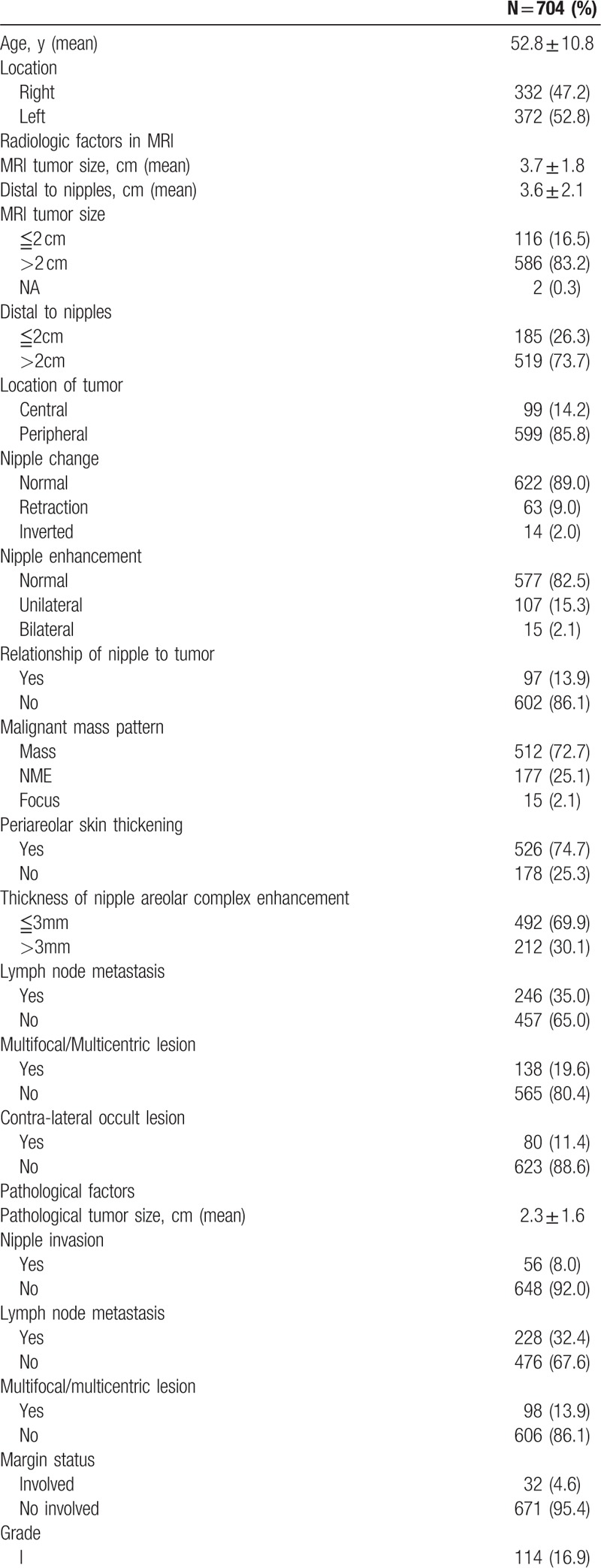
Clinical features of patients enrolled in MRI prediction of NAC invasion.

**Table 1 (Continued):**
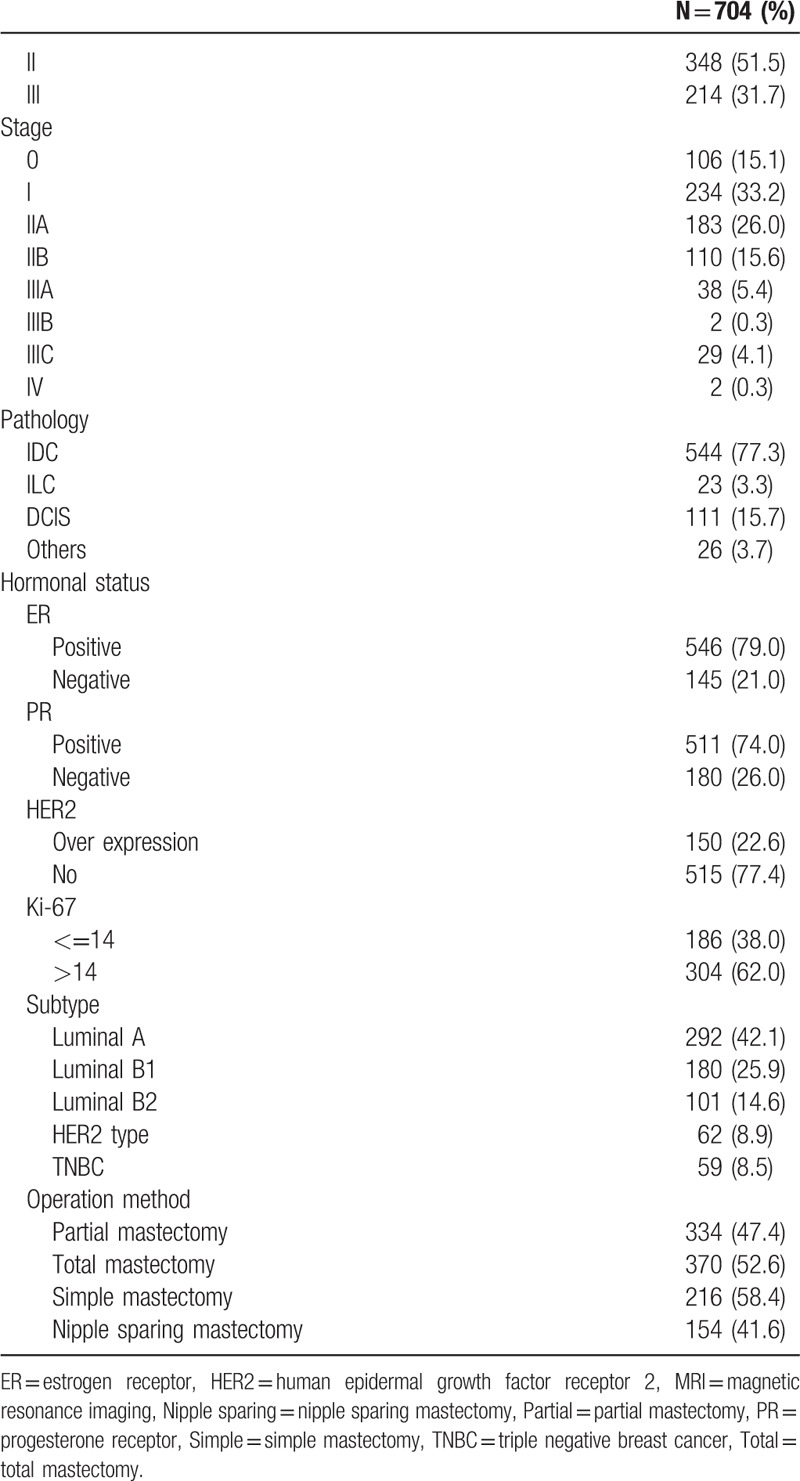
Clinical features of patients enrolled in MRI prediction of NAC invasion.

Of the MR image-related factors analyzed, we found that MRI tumor size, distance to nipples, location of tumor (central vs. peripheral), nipple change (retraction and/or inverted vs. normal), nipple enhancement (unilateral or bilateral vs. no enhancement), relationship of tumor to nipple (direct connection vs. no connection), and lymph node metastasis were predictive of NAC involvement (Table 2 ). Of the histopathological factors investigated, we found that pathologic tumor size, lymph node metastasis, grade, stage, estrogen receptor (ER) status, progesterone receptor (PR) status, human epidermal growth factor receptor 2 (HER2) status, and intrinsic tumor subtypes were associated with higher NAC involvement. Advanced age was also predictive of NAC involvement (Table 2 ).

**Table 2:**
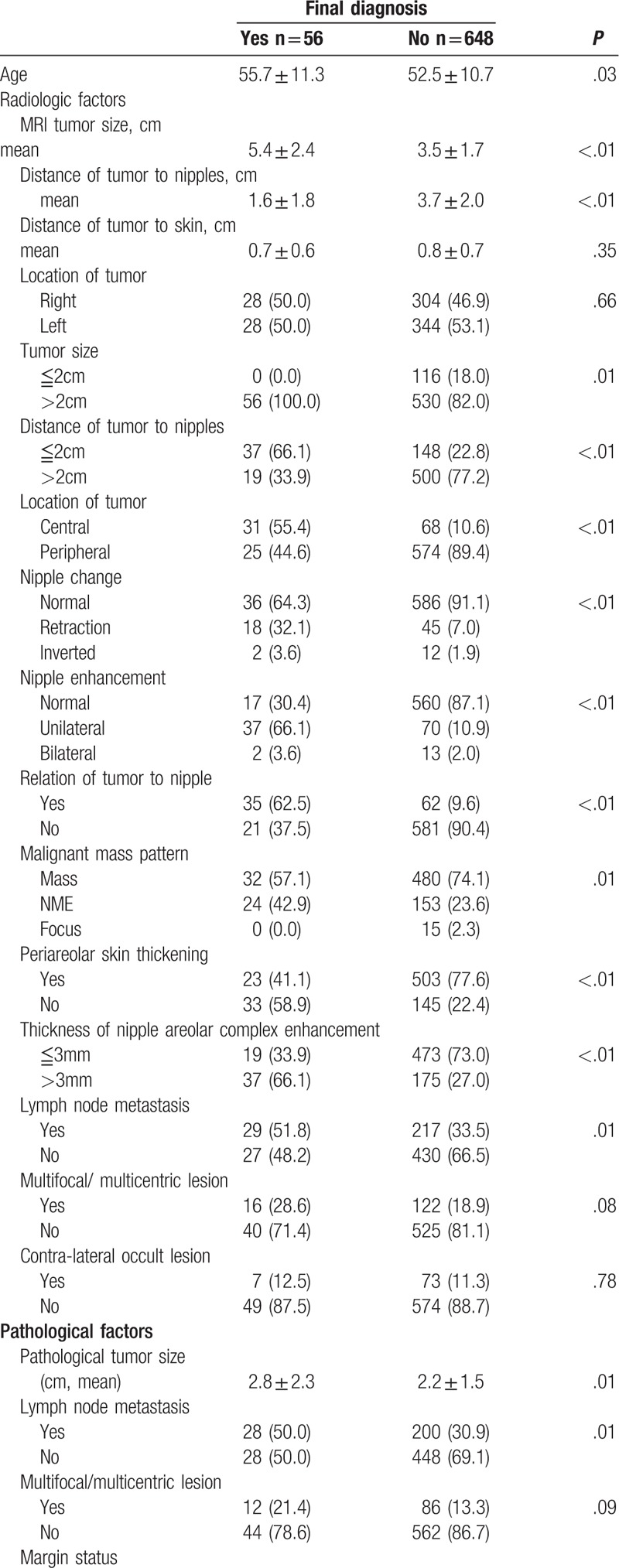
Clinicopathologic factors and radiologic features in MR images in patients with or without nipple invasion based on pathological diagnosis.

**Table 2 (Continued):**
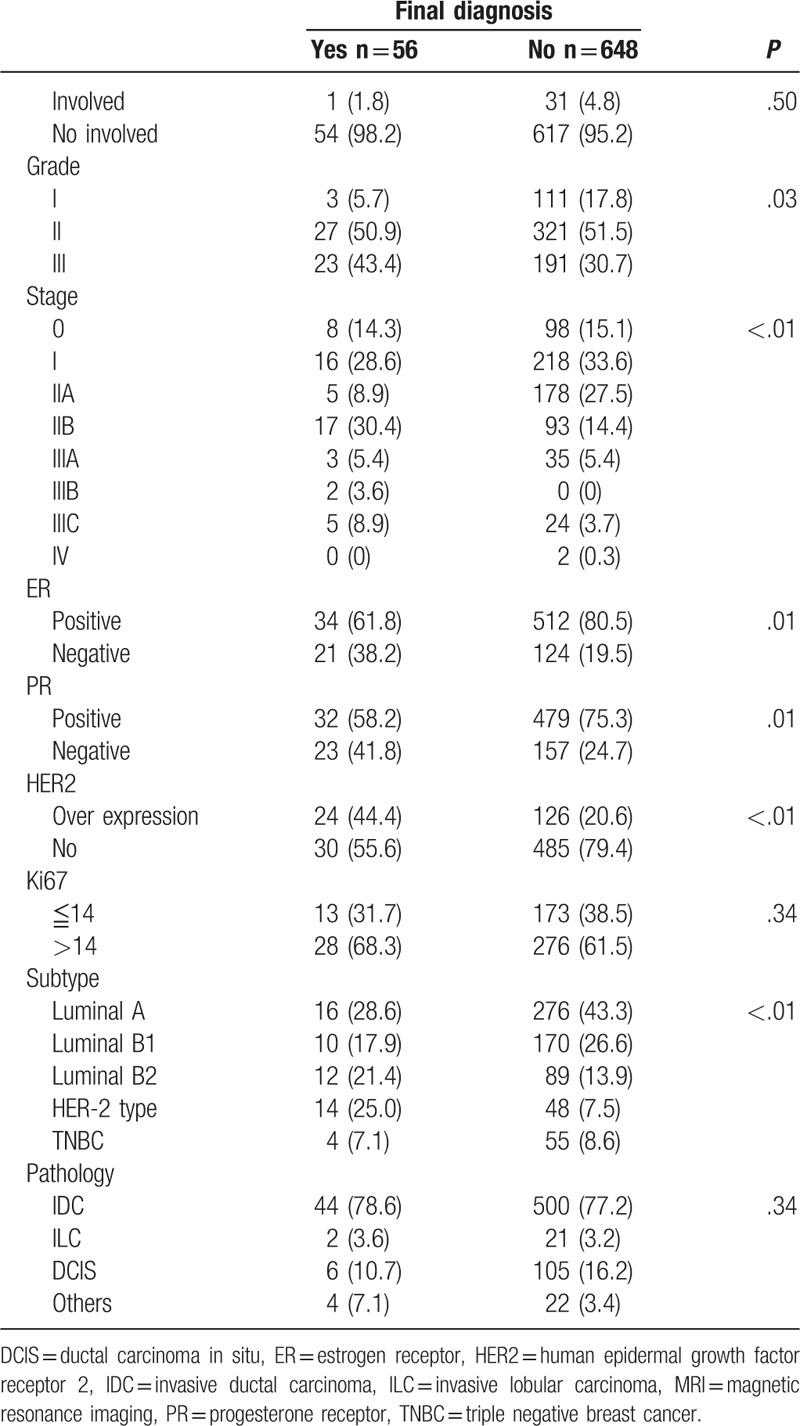
Clinicopathologic factors and radiologic features in MR images in patients with or without nipple invasion based on pathological diagnosis.

Results of the univariate and multivariate logistic regression analyses are presented in Table 3. The univariate analyses showed that tumor size (either derived from MR image or pathologic result), lymph node metastasis (as seen on MR images or as determined by histopathologic studies), central location of tumor, unilateral nipple enhancement, relationship of tumor to nipple, nipple change, malignant mass pattern, periareolar skin thickening, thickness of NAC enhancement, grade and hormone receptor (ER or PR) positive breast cancer were risk factors related to NAC invasion. We further examined the significant factors (*P* < .05) by multivariate analysis. Results of the multivariate analysis revealed that unilateral nipple enhancement (odds ratio = 4.86, 95% confidence interval [CI] 1.76–13.80, *P* ≤ .01), and pathologic lymph node metastasis (odds ratio = 2.43, 95% CI 1.16–5.18, *P* = .02) were the most significant independent predictors of NAC involvement (Table 3).

**Table 3:**
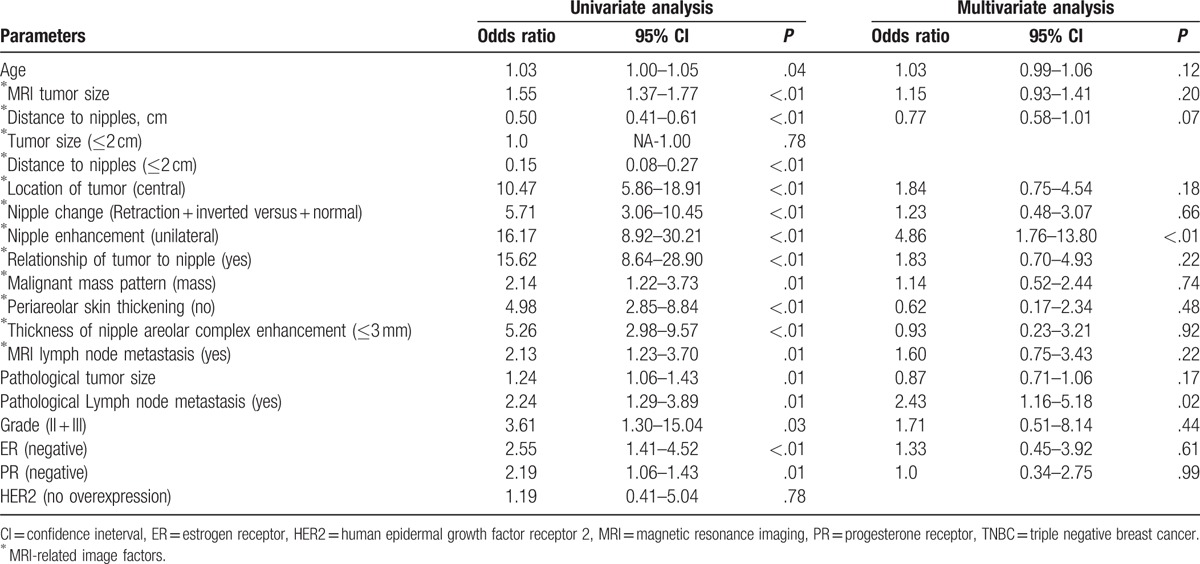
Risk factors for patients with nipple invasion based on pathological diagnosis.

To evaluate the diagnostic accuracy of MRI to diagnose the NAC invasion preoperatively, the images reading results of 2 independent radiologists (radiologist A and radiologist B) were compared with postoperative pathologic reports for concordance surveyed. Of the 159 patients who were judged by radiologist A to have evidence of NAC invasion on MR images before operation, 40 of them were found to have histopathologic evidence of NAC invasion at the subareolar region in final pathologic check-up (Fig. 2). According to the image to pathologic concordance results, the sensitivity of MRI to detect NAC invasion was 71.4% (40/56), the specificity was 81.6% (529/648), the PPV was 25.2% (40/159), the NPV was 97.1% (529/545) and the overall accuracy was 80.8% by radiologist A. In radiologist B, among the 121 patients suspected to have NAC invasion in MR images diagnosed preoperatively, 44 of them were found to have pathologic evidence of NAC invasion at the subareolar region. From the radiologist B’ reports, the sensitivity of MRI to detect NAC invasion was 78.6%, specificity was 88.1%, the PPV was 36.4%, the NPV was 97.9%, and the accuracy was 87.4%. The results of diagnostic accuracy of MRI to predict NAC invasion from the 2 individual radiologists were summarized in Table 4. The results of present study were compared with previous reported series and listed in Table 5.

**Table 4:**
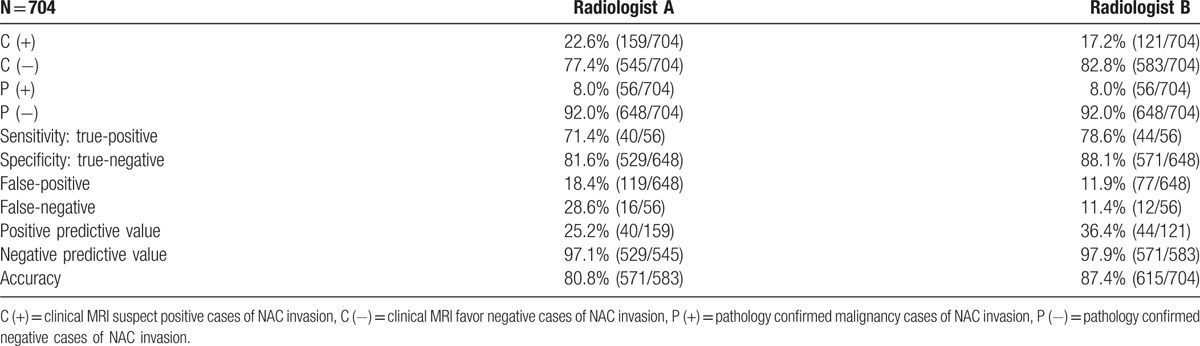
Diagnostic accuracy of breast MRI to predict NAC invasion between 2 different radiologists.

**Table 5:**
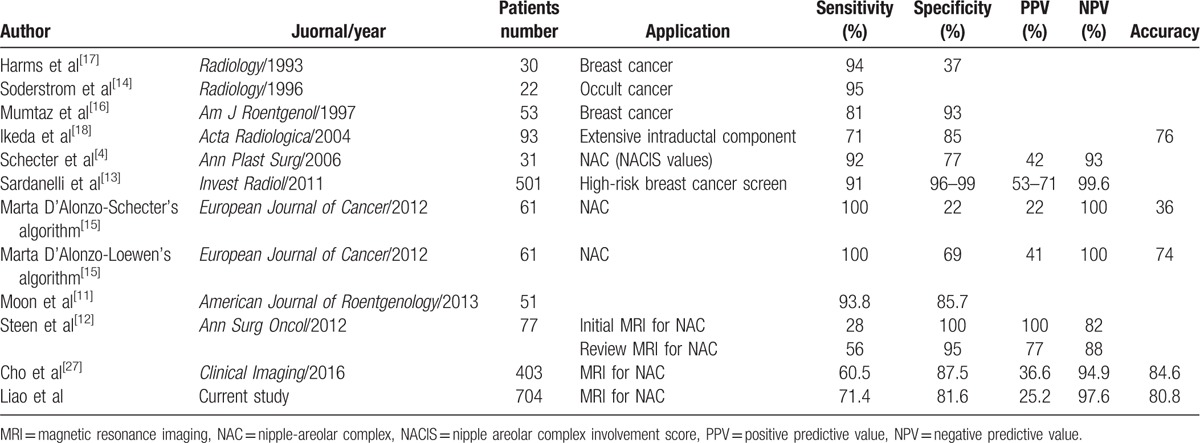
Literature review of MRI prediction of NAC invasion.

To evaluate the interobserver's variance and the positive predictive value of each potential predictor, we further evaluate the concordance between MRI NAC suspect invasion cases by Radiologist A, Radiologist B, and pathologic proven cases (Fig. 2). The number of suspect NAC invasion in MR images was 159 patients in Radiologist A, and 121 patients in Radiologist B. Fifty-six patients were found to have histologic evidence of cancer invasion at subareolar region (NAC invasion) by pathologist. The union of suspect NAC invasion in MR images in the 2 radiologists(C [+], clinical suspicious cases) and positive results in pathologist (P [+], pathologic proven cases) were total 207 patients (Fig. 2).

In regard to the MR evaluation of potential predictors between 2 radiologists, the 207 patients were further examined. The PPV (C[+] P[+]) in each potential predictor in Radiologist A ranged from 20.4% to 34.1% and Radiologist B ranged from 21.1% to 35.9% (Table 6).

**Table 6:**
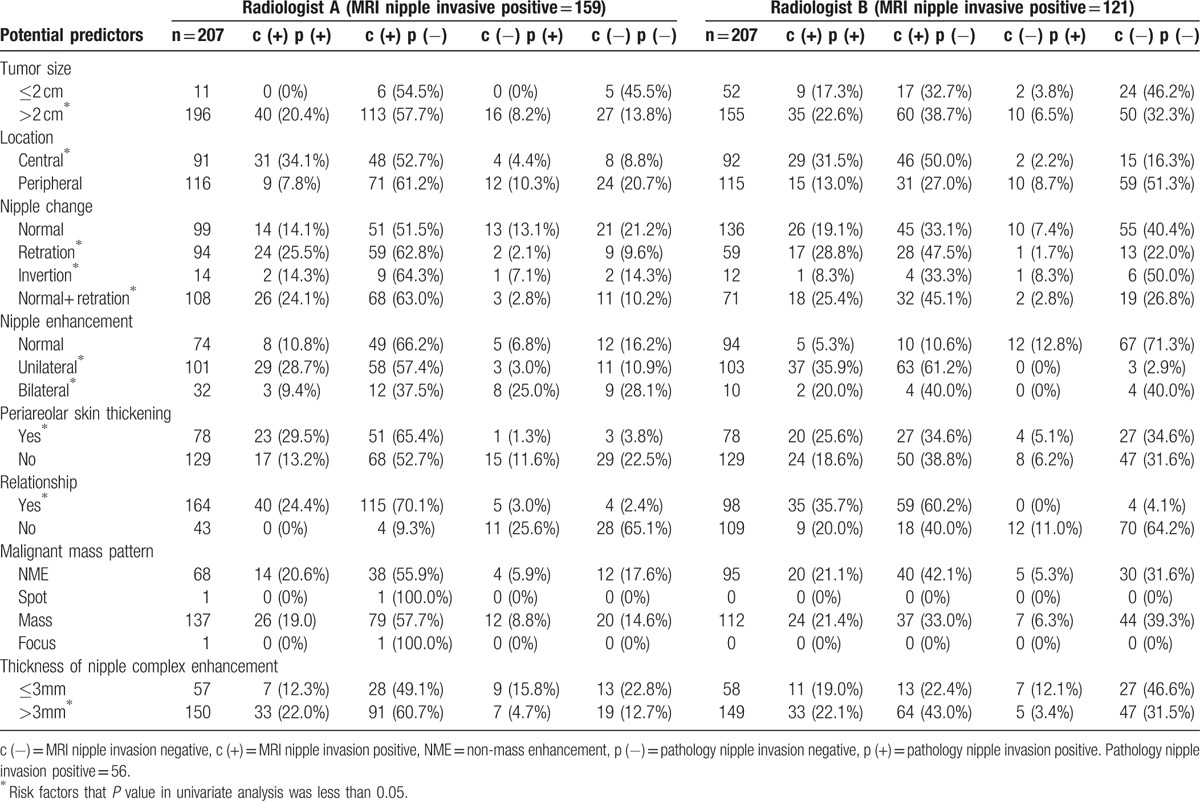
MR images evaluation of potential predictors between 2 radiologists.

Example of breast MRI figure and pathologic concordance of nipple areolar complex invasion was listed in Figure 3. The ROC curve test of 8 potential predictors of pathology nipple invasion, and inter-observers reliability of two radiologists were listed in Table 7. The area under curve (AUC) of each potential predictor in MR images derived from Radiologist A was ranged from 0.49 to 0.63, and Radiologist B ranged from 0.46 to 0.60. The final AUC of nipple invasion in MR images were 0.46 by Radiologist A and 0.64 by Radiologist B. From the above findings, one could say that all 8 predictors had poor accuracy in diagnosis of nipple invasion (Table 7). In regard to test the interobserver’ reliability, the interobserver kappa value of nipple invasion was −0.28, which represent the consistency between the radiologists was poor (Table 7).

**Table 7:**
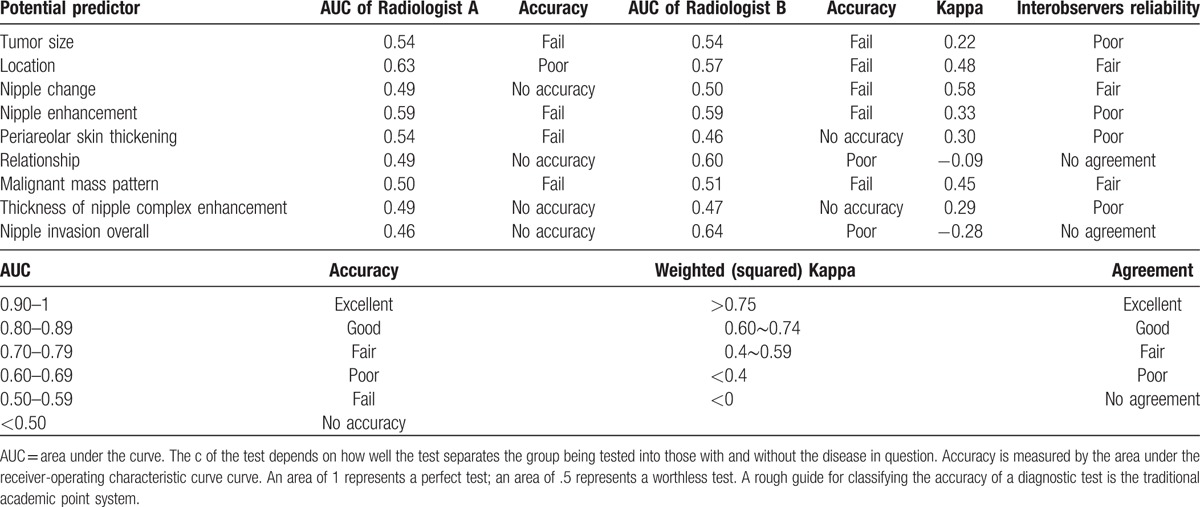
The receiver-operating characteristic curve test of potential predictors of pathology nipple invasion and interobservers’ reliability of 2 radiologists.

## 4 Discussion

We found that approximately 8.0% (56/704) of our study population had evidence of nipple invasion. Compared with 57 nonoperable breast cancer patients, the NAC invasion rate was significantly higher in nonoperable breast cancer patients (17.5% vs. 8%, *P* = .02, Fig. 1). These results were consistent with previously reported studies which revealed that nipple invasion ranges from 8% to 21%.^[19,20]^ In present study, clincopathologic factors like tumor size, lymph node metastasis, and central location of tumor were predictive of NAC invasion. These findings were consistent with previous reports, which showed that central tumor location,^[21,22]^ large tumor size (≧2 cm),^[23,24]^ nodal positivity,^[21,23,24]^ lymph vascular invasion,^[23,24]^ and multicentricity or multifocality^[21]^ were associated with nipple involvement.^[4–6,15]^ In a meta-analysis, tumor size and distance from the NAC were found to be the 2 main clinicopahtologic factors related to NAC involvement.^[5]^ Although tumor size is an important factor related to NAC invasion, preoperative mammography and/or ultrasound studies often underestimate the actual pathologic tumor size.^[25]^ Furthermore, occult nipple involvement by in situ carcinoma or Paget disease is very difficult to detect by clinical or conventional imaging examination alone.^[7]^ Studies were emerging to evaluate the potential of using MRI to diagnose NAC invasion preoperatively.^[11,12]^

In present study, MRI factors of tumor size, location, nipple change, nipple enhancement, periareolar skin thickening, relationship, malignant mass pattern, and thickness of NAC enhancement were potential predictors of nipple invasion (Tables 2 and 3 ). A number of studies have evaluated the usefulness of MRI in the assessment of nipple involvement.^[11,15,26,27]^ Sakamoto et al found that various enhancements on MRI, such as unilateral skin, periductal, rim shape, or segmental clumped enhancement were associated with histopathologic evidence of NAC invasion. Continuous enhancement from the index lesion to the nipple was an important predictor of nipple involvement.^[26,27]^ Other abnormal nipple morphology in MRI, such as thickness,^[27]^ bulkiness, and loss of normal tissue planes were highly specific of NAC involvement.

In our study, unilateral nipple enhancement (odds ratio = 4.86, 95% CI 1.76–13.80, *P* ≤ .01, Table 3) on MR images was the most significant independent image predictor of NAC involvement. Lee et al^[28]^ found that MR images displaying inhomogeneous and diffuse enhancement in areas of thickened skin and the parenchyma of the NAC were indicative of NAC invasion. Heywang et al found that abnormal nipple enhancement with an ill-defined thickened NAC were important factors related to NAC involvement. The enhancement of skin and NAC as seen in breast MRI was also an important clue for detection of local recurrence of cancer. Moon et al^[11]^ in their multivariate logistic regression analysis for pathologic diagnosis of NAC involvement showed that NAC enhancement, and NAC enhancement thickness were the 2 most important factors related to NAC invasion (*P* < .001).

Varied sensitivity and specificity of MRI to predict NAC invasion had been reported.^[4,11–18,27]^ Based on our literature review results in Table 5, the sensitivity of breast MRI ranged from 28% to 100%, and specificity ranged from 22% to 100%.^[4,11–18,27]^ From our preliminary results, the sensitivity reported by the principal investigator was 71.4% whereas that reported by the second investigator was 78.6%. Likewise, specificity was 81.6% versus 88.1%, and the accuracy was 80.8% versus 87.4%. The results seemed that the consistency between our 2 individual radiologists were very similar (Table 4). However, in the interobservers’ variance analysis, one could found that the kappa value of NAC invasion between 2 radiologists who evaluated the MR images in this study was only −0.28 (poor) (Table 7). When we further evaluated the consistency of images readings regarding each MRI potential predictors between 2 radiologists (Table 6), the kappa value ranged from −0.09 to 0.58 (Table 7), which revealed that there is substantial discrepancy between 2 individual radiologists in the interpretation of each MRI factors and subjective qualitative opinion of NAC invasion. These findings could explain why there is such a wide variation of sensitivity and specificity between different reported series about the application of MRI in the diagnosis of NAC invasion preoperatively (Table 5).

The PPV of breast MRI in our study ranged from 25.2% to 36.4%, which was consistent with the reported 22.0% to 44.0% range,^[4,15,27]^ but lower than 57% to 100%^[13]^ of PPV reported in the literature (Table 5). We hypothesized that the lack of objective diagnostic criteria may be a factor related to the wide discrepancy of MRI accuracy in the diagnosis of NAC invasion. From Table 6 and Table 7, we could find that each individual factors (tumor size, tumor-nipple distance, nipple inversion or retraction, periareolar skin thickening, NAC enhancement, relationship to the subareolar mass, malignant mass pattern, and thickness of NAC enhancement) had modest PPV (C[+] P[+]) ranged from 20.4% to 35.9% (Table 6), and AUC from 0.46 to 0.63 (Table 7), which indicated that neither factor was powerful enough to be a determinant diagnostic criteria for NAC invasion. The radiologist should coordinate with several predictors and/or using personal subjective qualitative opinion to diagnose NAC invasion. These findings could explain why MRI had low PPV and existed a wide discrepancy between different reported series (Table 5).

From our present study, the PPV of breast MRI ranged from 25.2% to 36.4%. In other words, the false-positive rate (FPR) ranged from 63.6% to 74.8%. The low PPV and a high FPV of MRI in predicting malignant invasion of NAC would be troublesome for preoperative decision-making as more aggressive surgical treatment such as total mastectomy (with NAC excised) would be suggested based on the MRI result. It would lead to a problem that most patients with so-called positive MR findings for NAC invasion will be overtreated surgically. Breast MRI as a potential powerful image tool can provide image evaluation different than mammogram and ultrasound. However, as NAC invasion might be because of some benign process such as infection or inflammation. It would be suggested that MRI study should not be the only criteria to diagnose tumor invasion to NAC. The high FPR should be improved for breast MRI to be widely used in the diagnosis of NAC invasion. To prevent overtreat in MR images positive patient, it was suggested to perform subnipple biopsy^[29]^ to confirm the malignant invasion of NAC during operation if preoperative MRI image suspect NAC invasion, whereas clinical presentation did not favor NAC invasion by cancer. In contrast to the low PPV, we found that MRI had very high NPV (reported 97.1%–97.9% in our study, and ranged from 82.0%–100% in literature review, Table 5). This high and reliable NPV of MRI could be of important value for preoperative surgical planning for patients selected for NSM if no sign of NAC invasion in MR images.

Our study is limited in its retrospective nature and possible selection bias. To evaluate the diagnostic accuracy of MRI in the prediction of NAC, we retrospectively enrolled 704 patients with preoperative MRI study and detailed pathologic report in single institution across 3 years. To conduct this image-pathologic concordance study, we did review all the imaging studies of these 704 cases; however, the pathologic samples were not rechecked again, but only pathology reports were examined. This can result in some bias as the pathology is the criterion standard in such image-pathologic concordance study. The difficulty of re-check subareolar region invasion in present study is that 334 (47.4%) of the 704 patients received only partial mastectomy, and 154 of the 370 mastectomy patients received NSM. Among the 334 of patients who received partial mastectomy, only 5 (1.5%) of them received excision of the NAC owing to NAC invasion. The other 329 partial mastectomy patients were assumed that there were no pathologic subareolar invasion. Owing to these reasons, we could not have the subareolar (defined as ≤1 cm from the base of nipple) pathologic reviews in our whole study population. Thus, the pathologic proven 56 (8%) cases in our study might be underestimated as the malignant subareolar region invasion could not be actually re-evaluated by pathologists. This could be another factor that lowers the PPV of MRI in our current study.

In conclusion, MRI showed acceptable accuracy, and impressive NPV but low PPV in evaluation of malignant NAC invasion preoperatively. MRI finding of unilateral nipple enhancement was the most significant predictor of NAC involvement. MR images are useful in initial evaluation, and patients with no sign of NAC invasion were good candidates to preserve of NAC. However, when MR images suspect positive nipple invasion, a further confirmation with sub-nipple biopsy may be needed in clinical no apparent NAC involved cases. Presently, MRI study could not be the only criteria for diagnose malignant invasion of NAC to prevent over treat surgically.
