# Role of breast magnetic resonance imaging in predicting malignant invasion of the nipple-areolar complex: Potential predictors and reliability between inter-observers: Erratum

**DOI:** 10.1097/MD.0000000000007725

**Published:** 2017-07-28

In the article, “Role of breast magnetic resonance imaging in predicting malignant invasion of the nipple-areolar complex: Potential predictors and reliability between inter-observers”,^[1]^ which appeared in Volume 96, Issue 28 of *Medicine*, Dr. Chiung-Ying Liao's named appeared incorrectly as Chun-Ying Liao.
